# Suppression of Adenosine-Activated Chloride Transport by Ethanol in Airway Epithelia

**DOI:** 10.1371/journal.pone.0032112

**Published:** 2012-03-19

**Authors:** Sammeta V. Raju, Guoshun Wang

**Affiliations:** 1 Department of Genetics, Louisiana State University Health Sciences Center, New Orleans, Louisiana, United States of America; 2 Department of Microbiology, Louisiana State University Health Sciences Center, New Orleans, Louisiana, United States of America; 3 Department of Medicine, Louisiana State University Health Sciences Center, New Orleans, Louisiana, United States of America; Virginia Commonwealth University, United States of America

## Abstract

Alcohol abuse is associated with increased lung infections. Molecular understanding of the underlying mechanisms is not complete. Airway epithelial ion transport regulates the homeostasis of airway surface liquid, essential for airway mucosal immunity and lung host defense. Here, air-liquid interface cultures of Calu-3 epithelial cells were basolaterally exposed to physiologically relevant concentrations of ethanol (0, 25, 50 and 100 mM) for 24 hours and adenosine-stimulated ion transport was measured by Ussing chamber. The ethanol exposure reduced the epithelial short-circuit currents (I_SC_) in a dose-dependent manner. The ion currents activated by adenosine were chloride conductance mediated by cystic fibrosis transmembrane conductance regulator (CFTR), a cAMP-activated chloride channel. Alloxazine, a specific inhibitor for A_2B_ adenosine receptor (A_2B_AR), largely abolished the adenosine-stimulated chloride transport, suggesting that A_2B_AR is a major receptor responsible for regulating the chloride transport of the cells. Ethanol significantly reduced intracellular cAMP production upon adenosine stimulation. Moreover, ethanol-suppression of the chloride secretion was able to be restored by cAMP analogs or by inhibitors to block cAMP degradation. These results imply that ethanol exposure dysregulates CFTR-mediated chloride transport in airways by suppression of adenosine-A_2B_AR-cAMP signaling pathway, which might contribute to alcohol-associated lung infections.

## Introduction

Alcohol abuse is a risk factor for pulmonary infections. It is not fully understood how alcohol exposure compromises the lung host defense. Previous studies suggest that multiple pathophysiological mechanisms may be involved [1], [2], [3]. Airway mucosal immunity and mucociliary clearance are the two primary host defense mechanisms, which take place in a thin layer of liquid on the top of airway epithelia, known as airway surface liquid (ASL). ASL, composed of a gel-like mucus layer and a sol-like periciliary liquid layer [4], [5], [6], is the “battlefield” for pulmonary infection and immunity. The viscous mucous blanket traps inhaled microorganisms and particles to restrict their spreading in the lung. In contrast, the watery periciliary liquid (PCL) underneath pools antimicrobial substances, antibodies, cytokines, chemokines and other immune modulators [7], [8], [9]. More importantly, PCL provides the milieu for innate and adaptive immune cells including neutrophils and macrophages to home and function. Moreover, PCL prevents cilia from being entrapped in viscous mucus and bathes them for mechanical movement for mucociliary clearance [5], [10].

ASL composition and volume are collectively regulated by epithelial chloride secretion, sodium absorption and secondarily water secretion and absorption [6], [11]. Mounting evidence indicates that paracrine/autocrine purinergic signaling is critical to airway epithelial ion transport and ASL hydration [12]#. Adenosine has been shown to be a potent regulator in the process, which can be directly released by local epithelial cells and immune cells [13] or from extracellular metabolism of ATP [12]#. It is known that ATP is constitutively released by epithelia due to various stimuli including mechanical stretch and shear stress due to respiration [14]. The released ATP is then converted to adenosine by extracellular ectonucleotidases [15]. Thus, ASL has relatively high levels of adenosine. Further studies demonstrate that adenosine largely regulates epithelial CFTR channel function by acting on A_2B_AR [16], [17], [18]. Thus, the adenosine-A_2B_AR signaling pathway is a crucial element in lung host defense [19], [20].

Previous alcohol studies have documented that ethanol exposure decreases cAMP signaling and protein kinase A (PKA) activation [3], [21]. Ethanol also up-regulates phosphodiesterase 4 (PDE4), which increases cAMP degradation [22]#. In spite of the clear link between alcohol exposure and alteration of adenosine signaling, no published data are currently available concerning alcohol effects on airway ion transport through this signaling pathway. The current report directly measured the adenosine-induced chloride secretion of airway epithelia under the exposure of physiologically relevant concentrations of alcohol and found that ethanol attenuates epithelial CFTR-mediated chloride transport by modulating cellular cAMP levels.

## Materials and Methods

### Ethics statement

No human subjects or animals were used in this study.

### Cell culture

Calu-3 cells, a human airway epithelial cell line (ATCC, Manassas, VA), were seeded on collagen-coated Millicell®-PCF membrane inserts (Millipore, Billerica, MA) at a density of 1×10^6^ cells per insert of 0.6 cm^2^ surface area. Two days after the initial submerged culture, the apical media were aspirated off and the cells cultured at an air-liquid interface according to previously published protocol [23]. Regardless of submerged culture or air-liquid interface culture, the media used were the same, consisting of Advanced-MEM (Gibco, Carlsbad, CA) containing 10% fetal bovine serum, 1% L-glutamine, 100 U/ml penicillin, 100 µg/ml streptomycin and 0.25 µg/ml amphotericin B. After 2 weeks at 37°C in presence of 5% CO_2_, the epithelia established a dry apical surface and had a transepithelial electrical resistance greater than 1000 Ω/cm^2^. The cystic fibrosis (CF) airway epithelial cells CFBE41o- [24] were similarly cultured. The fully differentiated CF epithelia after 2 weeks exhibited a transepithelial resistance greater than 700 Ω/cm^2^.

### Ethanol exposure

Air-liquid interface cultures were basolaterally exposed to different concentrations of ethanol (200 Proof; AAPER Alcohol and Chemical Co., Shelbyville, KY), as indicated in individual experiments. All the cultures were kept at 37°C, 5% CO_2_ in incubators that had been pre-saturated with specified concentrations of ethanol.

### Transepithelial electrical resistance (TEER) measurement

The TEER of the airway epithelial cell cultures was measured by using a “chop stick” epithelial ohmmeter (World Precision Instruments, Sarasota, FL), as described previously [25], [26].

### Airway Epithelial Electrophysiology

Calu-3 cells, cultured at the air-liquid interface for 2 weeks, were exposed to different concentrations of ethanol for 24 hours and mounted on an Ussing chamber apparatus (World Precision Instruments, Sarasota, FL). The cells were bathed with an apical low chloride buffer (135 mM sodium gluconate, 5.0 mM HEPES, 1.2 mM MgCl_2_, 0.6 mM KH_2_PO_4_, 4 mM CaCl_2_, 2.4 mM K_2_HPO_4_ ˙3H_2_O, and 10 mM Dextrose, pH 7.4) and with a basal high chloride buffer (135 mM NaCl, 5.0 mM HEPES, 1.2 mM MgCl_2_, 0.6 mM KH_2_PO_4_, 1.2 mM CaCl_2_, 2.4 mM, K_2_HPO_4_ ˙3H_2_O, and 10 mM Dextrose, pH 7.4). Both buffers were continuously stirred, gassed with 95% O_2_ and 5% CO_2_ and maintained at 37°C. These buffers were to maintain a chloride gradient across the epithelial monolayer. The TEER was measured with an open circuit by applying an electrical pulse across the epithelial monolayer. Short circuit currents (I_SC_) were measured by applying an epithelial voltage clamp. For all the experiments, 100 µM of amiloride was added to the apical side to block sodium channels. Anion currents were induced by apical addition of 100 µM adenosine. The A_2B_AR blockade was achieved by apical application of 50 µM alloxazine. To differentiate between chloride and bicarbonate currents, 100 µM bumetanide was used basally to block chloride transport. Acetazolamide (20 µM) or DNDS (4,4′-dinitro stilbene-2,2′-disulfonate) (100 µM) was employed apically to block bicarbonate transport. Further, to inhibit epithelial phosphodiesterases, 100 µM of IBMX (3-isobutyl-1-methylxanthine) or 50 µM papaverine was added apically.

### Intracellular cAMP Measurement

The air-liquid interface cultures of Calu-3 cells were exposed to either 0 mM or 100 mM of ethanol at the basolateral side for 24 hours. On the apical surface the cells were stimulated with 100 µM of adenosine. The cells were washed with PBS containing 100 µM IBMX to inhibit phosphodiesterases that might degrade cAMP. The cells were lysed and cAMP was measured by cAMP immunoassay (R&D Systems, Minneapolis, MN). IBMX (100 µM) was also included in the lysis buffer. The samples were read at 450 nm using a spectrophotometer (BioTEK, Winooski, VT). All the treatments were carried out in quadruplicates.

### Statistical Analysis

Data presented represent mean of multiple experiments and error bars indicate standard deviation from the mean. Where indicated, the data points were analyzed by Student's t-test or One-way ANOVA test. The *P* values smaller than 0.05 were considered statistically significant.

## Results

### Ethanol suppresses adenosine-activated ion transport by airway epithelia

**Figure 1:**
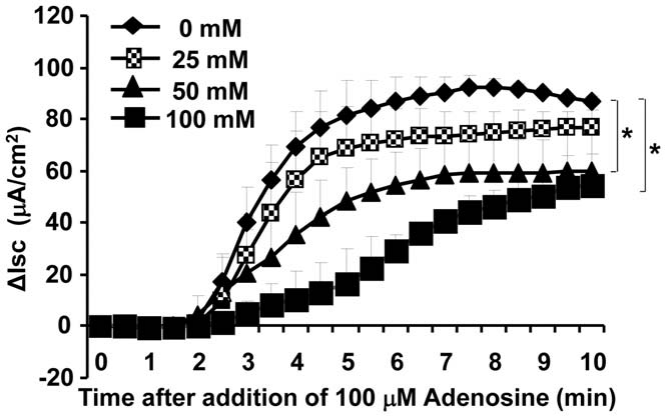
Effect of ethanol pre-exposure on adenosine-induced epithelial ion transport. Calu-3 cells were cultured at an air-liquid interface on membrane filters and basolaterally exposed to 0, 25, 50 and 100 mM of ethanol for 24 hours. These Calu-3 epithelia were placed in an Ussing chamber with asymmetrical buffers of chloride (apical 10.4 mM and basolateral 139.8 mM). Following voltage clamp, stable short circuit baselines were attained in 15 to 20 min. I_SC_ was measured after blocking Na^+^ channels with 100 µM of apical amiloride and stimulated with 100 µM of apical adenosine. Alterations in ion transport are expressed as difference in I_SC_ from their baseline. Ethanol pre-exposure decreased 100 µM adenosine mediated epithelial ion transport in a dose-dependent manner. Asterisks indicate significant differences between groups by One-way ANOVA test (*p*<0.05, n = 5 for each condition).

To explore if ethanol affects adenosine-activated ion transport function of airway epithelium, we employed air-liquid interface cultures of Calu-3 cells, a system widely used to investigate airway epithelial electrophysiological properties [27], [28]. The cultured epithelia were exposed basolaterally for 24 hours to different concentrations of ethanol (0, 25, 50 and 100 mM) and adenosine-induced transepithelial ion transport was assessed by measuring I_SC_ with an Ussing chamber apparatus. Two buffers with asymmetric chloride were applied: apical low chloride (10.4 mM) and basolateral high chloride (139.8 mM). After voltage clamp, sodium channels were blocked by apical amiloride (100 µM) and I_SC_ was stimulated by apical addition of adenosine (100 µM). As shown (**Fig. 1**), the ethanol exposure decreased adenosine-activated I_SC_ in a dose-dependent manner. Significant differences were detected by One-way ANOVA test between the control and the alcohol-exposed (50 mM and 100 mM) groups (*p*<0.05, n = 5).

### Ethanol suppresses adenosine-activated chloride transport

**Figure 2:**
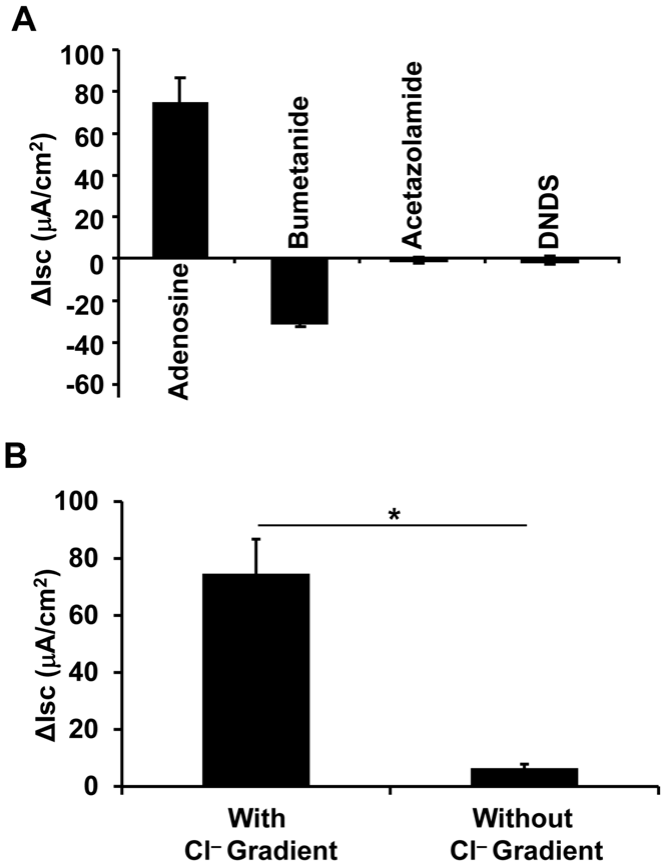
Adenosine-stimulated short circuit current is chloride conductance. **A**) I_SC_ of Calu-3 epithelia was measured by using the asymmetric chloride buffers. After stimulated with 100 µM of apical adenosine, the cells gave rise to I_SC_ of ∼75 µA/cm^2^. The I_SC_ was blocked by ∼58% by basolateral addition of bumetanide (100 µM), but not by acetazolamide (20 µM) or DNDS (100 µM). **B**) I_SC_ of Calu-3 epithelia was measured with either asymmetric chloride buffers or symmetric chloride buffers. The adenosine-stimulated I_SC_ with no chloride-gradient buffers was ∼92% lower than that with chloride-gradient buffers. Significance of the difference was determined by Student's t-test (*p*<0.01, n = 5).

It was previously documented that adenosine-stimulated anion secretion in airway epithelia is largely mediated by chloride transport [17], [29]. To explore if the alcohol-suppressed I_SC_, as identified above, is actually a chloride current, we first confirmed chloride transport by Calu-3 epithelia in our experimental setting. To this end, the cultured Calu-3 epithelia without any alcohol exposure were subjected to I_SC_ measurement in the presence of various inhibitors to block different ion channels. Bumetanide (100 µM), a specific inhibitor for the Na^+^-K^+^-2Cl^−^ cotransporter, was applied to the basolateral side of the Calu-3 epithelia after adenosine stimulation. This drug decreased I_SC_ by ∼58%, while acetazolamide, a carbonic anhydrase inhibitor, and DNDS, an inhibitor for Na^+^/HCO_3_^−^ cotransporters and Cl^−^/HCO_3_^−^ exchangers, had no effect on I_SC_ (**Fig. 2A**). These data suggest that the adenosine-stimulated anion current that is suppressed by alcohol is the chloride channel conductance. The result was further validated by measuring the adenosine-stimulated I_SC_ with identical apical and basal chloride buffers. Without chloride gradient (**Fig. 2B**), adenosine-induced I_SC_ was dropped significantly by Student's t-test (*p*<0.01, n = 5). The I_SC_ was only ∼8% of that measured with the asymmetric chloride buffers.

### The adenosine-stimulated chloride transport is mediated through the CFTR channel

**Figure 3:**
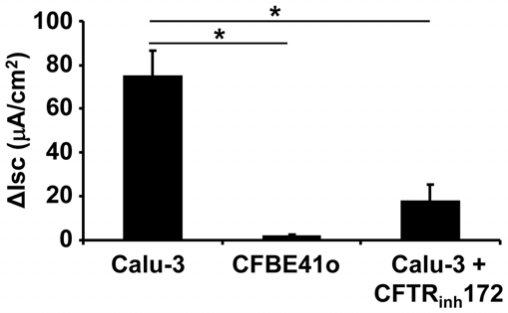
Adenosine-stimulated transepithelial I_SC_ was mediated by CFTR channel. Calu-3 epithelia were stimulated with 100 µM of apical adenosine, resulting in a significant increase in I_SC_ above baseline. Such an adenosine-induced I_SC_ was mostly absent in CFBE41o cells in which CFTR channel was dysfunctional or was significantly inhibited with 25 µM of CFTR inhibitor CFTR_inh_172. Student's t-test was performed to determine the statistic significance (*p*<0.05, n = 5).

CFTR has been found to be a major chloride channel in the airway epithelium responsible for adenosine-induced ion transport [30]#. To validate if CFTR is responsible for the observed adenosine-stimulated chloride transport in the Calu-3 epithelia, we applied CFTR channel inhibitor CFTR_inh_172 to the apical side of Calu-3 epithelia for 30 minutes, followed by adenosine stimulation. The chloride conductance was decreased by ∼76% when CFTR was inhibited (**Fig. 3**), which is significantly lower than that of the no drug control (*p*<0.01, n = 5). To seek a second approach to confirm the result, CFBE41o cells, an airway epithelial cell line derived from a cystic fibrosis (CF) patient with homozygous ΔF508 mutations in CFTR, were used [24]. Strikingly, adenosine failed to elicit any chloride currents across the CF epithelia (*p*<0.01, N = 4). Thus, the currents under our experimental condition and drug profile are CFTR-mediated chloride conductance. These data altogether confirmed that in airway epithelial cells ethanol-suppression of chloride transport is mediated through CFTR, a cAMP-activated chloride channel.

### A_2B_AR predominantly regulates the adenosine-induced chloride secretion in Calu-3 cells

**Figure 4:**
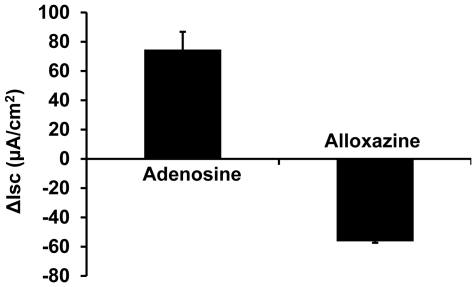
Adenosine stimulates chloride conductance through the A_2B_ adenosine receptor (A_2B_AR). The air-liquid interface cultures of Calu-3 cells were stimulated with 100 µM of apical adenosine, followed by apical addition of 50 µM of alloxazine, a specific inhibitor for A_2B_AR. The adenosine-stimulated I_SC_ was decreased by ∼75% (n = 6).

The aforementioned data suggest that the negative modulation of epithelial chloride secretion by ethanol is likely through the adenosine-adenosine receptor signaling pathway. Up to date, four different adenosine receptors have been identified: A1, A_2A_, A_2B_ and A3 receptors [31]. In airway epithelial cells, A_2B_AR predominantly regulate CFTR function [18]. Here, we wanted to examine if the same signaling pathway is involved in the alcohol-induced inhibition of CFTR-mediated chloride transport. To this end, alloxazine (50 µM), a commonly used A_2B_AR specific blocker, was applied to the apical buffer after adenosine stimulation. The data (**Figure 4**) demonstrate that ∼75% adenosine-stimulated epithelial I_SC_ was reduced by alloxazine, indicating that A_2B_AR was largely responsible for the adenosine-mediated chloride secretion.

### Ethanol exposure affects CFTR-mediated chloride secretion through modulation of cellular cAMP level

**Figure 5:**
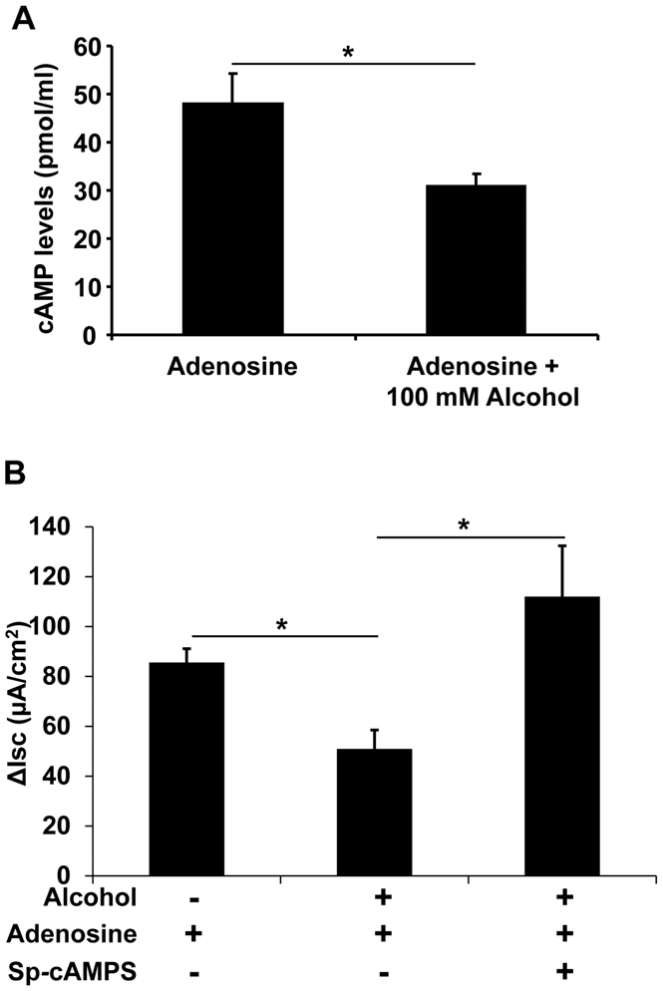
Effect of ethanol exposure on cellular cAMP level and adenosine analog antagonizes ethanol suppression of adenosine-induced chloride secretion. **A**) Calu-3 epithelia, exposed to 0 and 100 mM of ethanol for 24 hours, were apically stimulated with 100 µM of adenosine for 10 min. Cells were lysed and cAMP levels were estimated by immunoassay. Ethanol pre-exposure significantly decreased the adenosine-induced cellular cAMP level to ∼64% of the no ethanol control (*p*<0.05, n = 5). **B**) In Calu-3 epithelia, ethanol pre-exposure decreased the adenosine-stimulated I_SC_. Such an inhibitory effect of ethanol on adenosine-induced I_SC_ was antagonized by Sp-cAMPS, a phoshodiesterase-resistant cAMP analog (*p*<0.05, n = 6).

Upon adenosine binding, the A_2B_AR signals through G_s_ protein to activate adenylyl cyclase and raises intracellular cAMP [13], [32]. Because CFTR is a cAMP-activated chloride channel, we hypothesized that ethanol inhibits adenosine-stimulated cAMP production to cause a reduced CFTR channel activity. To test this hypothesis, air-liquid interface cultures of Calu-3 cells were exposed basolaterally to either 0 mM or 100 mM of ethanol for 24 hours. These cells were apically stimulated with 100 µM of adenosine as described above for 10 minutes, the time point at which the epithelial I_SC_ was at its peak levels. Then, the cells were lysed and the levels of cellular cAMP assayed. The results demonstrate that ethanol pre-treatment significantly decreased adenosine-stimulated cAMP levels (*p*<0.05, n = 5) (**Fig. 5A**). In addition, to test whether the decrease in cAMP was in fact causing the suppression of adenosine-stimulated chloride transport by ethanol, Sp-cAMPS, a cell permeable and phosphodiesterase-resistant cAMP analogue, was used to directly activate PKA which then activates the CFTR channel. As shown in **Figure 5B**, the alcohol-treated Calu-3 cells in the presence of 10 µM Sp-cAMPS obliterated the ethanol suppressive effect on adenosine-induced chloride secretion (*p*<0.05, n = 6). Thus, we conclude that ethanol inhibits CFTR-mediated chloride secretion by directly affecting the cellular cAMP level instead of the downstream PKA enzyme.

### Ethanol-suppression of chloride secretion in airway epithelial cells can be restored by phosphodiesterase inhibitors

**Figure 6:**
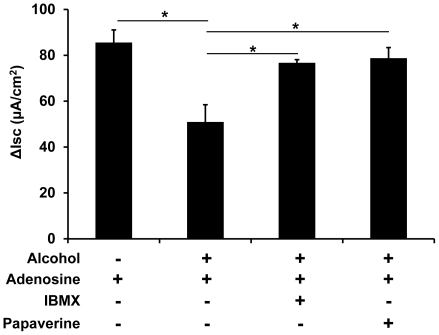
Phosphodiesterase inhibitors restore the ethanol suppression of adenosine-induced transepithelial chloride conductance. The air-liquid interface cultures of Calu-3 cells were exposed to 0 and 100 mM of ethanol for 24 hours. Adenosine-stimulated I_SC_ was measured, showing that ethanol-exposed Calu-3 epithelia had significantly lower adenosine-stimulated I_SC_ than the no ethanol control. However, phosphodiesterase inhibitors, IBMX (100 µM) or papaverine (50 µM), fully restored the ethanol-suppressed adenosine-stimulated I_SC_. Asterisks indicate statistically significant differences between groups (p<0.05, n = 4 for each group).

Based on the data that ethanol impairs the adenosine-activated chloride secretion by reducing the cellular cAMP level, we chose to block endogenous cAMP degradation pharmacologically to counteract the alcohol-suppressive effect on chloride secretion. Air-liquid interface cultures of Calu-3 cells were similarly exposed to ethanol for 24 hours and treated with 100 µM of non-specific phosphodiesterase inhibitor IBMX along with adenosine stimulation. The data in **Figure 6** indicate that IBMX almost completely restored the ethanol suppression of Calu-3 chloride secretion (*p*<0.05, n = 4). Papaverine is a clinically used phosphodiesterase inhibitor. This drug also overcame the inhibitory effect of ethanol on adenosine-induced transepithelial chloride conductance (p<0.05, n = 4) (**Fig. 6**). These results not only confirm that ethanol modulates adenosine-cAMP signaling but also suggest that phosphodiesterase inhibitors may be useful as the potential therapeutic agents for improving the airway epithelial ion transport and mucociliary clearance in alcoholic patients.

## Discussion

Airway epithelial cells not only constitute the physical barrier that separates airway lumen from interstitial compartments, but also actively participate in innate and adaptive immunity to protect the host from pulmonary infections [33]. Multiple defense systems have been evolved in airways. First, the polarized airway epithelia have a mechanical clearance mechanism. Goblet cells or submucosal glands secrete mucus that entraps airborne particles and inhaled infectious agents. Synchronized ciliary movement sweeps inhaled particulate matter toward the mouth to be expectorated or swallowed [34]#. Second, airway epithelia secrete antimicrobial factors, such as lysozyme and β-defensins [35]. Third, airways and alveoli are patrolled by phagocytic cells, most notably the alveolar macrophage, which can engulf microbes [36]. Fourth, airways can recruit neutrophils and monocytes to sites of inflammation [37]. Fifth, the acquired immune system, including antigen-stimulated T and B lymphocytes, provides cellular and humoral defenses for the airways [38], [39]. It is noteworthy that all the above-mentioned pulmonary defense mechanisms are executed in ASL, the thin film of liquid on top of the airway epithelia. Therefore, ASL homeostasis is pivotal to lung host defense. Here we provide the first evidence suggesting that ethanol exposure suppresses adenosine-stimulated chloride secretion that regulates ASL.

Our data demonstrate that ethanol affects adenosine-stimulated chloride secretion through CFTR. On epithelial apical surface, adenosine binds to A_2b_AR, a predominant adenosine receptor present in airway epithelia [16]. This receptor is coupled to G_s_ protein to activate adenylyl cyclase, which elevates intracellular cAMP and consequently activates PKA. Then, CFTR is phosphorylated by PKA and the channel opens to permeate chloride [40]#. More chloride in ASL will cause less Na^+^ absorption and more water retention, thus increasing ASL height and volume [15], [20]. It is even speculated that adenosine is a sensor for ASL homeostasis. When ASL falls, adenosine levels increase in airways beyond their basal levels and activate A_2B_AR and CFTR channels [16], [29]. Our data have linked ethanol-suppression of cAMP levels to attenuation of chloride secretion by airway epithelia. Previous studies have documented that ethanol decreases ciliary beating and epithelial cell migration during wound repair which are associated with compromised cAMP signaling and PKA activation [21], [41]. Moreover, alcohol is reported to upregulate the PDE4 enzyme expression as well as the enzymatic activity in epithelia [22] #, which results in accelerated cAMP degradation. Thus, our data are consistent with the data reported, indicating that alcohol modulates various cellular pathways by reducing cellular cAMP levels.

Pulmonary mucociliary clearance is largely affected by three factors: 1) mucus production, 2) ciliary sweeping and, 3) ASL state. Studies on human bronchial epithelial cells have revealed that a 24-hour exposure with 100 mM of alcohol caused an 8-fold increase in trachea-bronchial mucin gene expression [42]. Interestingly, experiments using bovine bronchial epithelial cells have established that alcohol exposure at 100 mM beyond 6 hours decreases ciliary beating frequency [22], [41]. A recent publication by Allen-Gipson and colleagues reports that purinergic stimulation of CBF requires A_2B_AR activation [43]. Similarly, in rats chronic alcohol administration decreased ciliary beating frequency and enhanced lung colonization of nasally administered *S. pneumoniae* [44] #. Our current results demonstrate that 24-hour ethanol exposure reduces chloride secretion, which consequently alters ASL composition and volume. Thus, alcohol affects all three mucociliary clearance components.

Pharmacologically, blocking cAMP degradation has the potential of restoring ethanol-suppressed cellular cAMP levels and therefore epithelial functions. This finding is of clinical implications. Phosphodiesterase inhibitors such as papaverine may improve mucociliary clearance in alcoholics by enhancing airway epithelial ion secretion and ciliary beating. Moreover, enhancement of airway epithelial ion secretion alters the composition of ASL. Our laboratory has reported that extracellular chloride levels affect neutrophil microbial killing ability [45]. Thus, increasing ASL chloride level by phosphodiesterase inhibitors may also improve phagocytic innate immunity in airways of alcoholics.

In summary, ethanol exposure compromises chloride ion secretion which is pivotal to maintain ASL volume and composition. Such an effect may alter the properties of airway host defenses predisposing ethanol abusers to an increased risk of infection in the lung. Restoration of cellular cAMP level with phosphodiesterase inhibitors could potentially ameliorate mucociliary clearance by improving epithelial ion secretion and hence lung host defense.
